# The potential of small-Unmanned Aircraft Systems for the rapid detection of threatened unimproved grassland communities using an Enhanced Normalized Difference Vegetation Index

**DOI:** 10.1371/journal.pone.0186193

**Published:** 2017-10-12

**Authors:** Conor J. Strong, Niall G. Burnside, Dan Llewellyn

**Affiliations:** 1 Ecosystem and Environmental Management Research Group, School of Environment & Technology, University of Brighton, Brighton, United Kingdom; 2 LDP LLC, Carlstadt, New Jersey, United States of America; Universidade de Aveiro, PORTUGAL

## Abstract

The loss of unimproved grassland has led to species decline in a wide range of taxonomic groups. Agricultural intensification has resulted in fragmented patches of remnant grassland habitat both across Europe and internationally. The monitoring of remnant patches of this habitat is critically important, however, traditional surveying of large, remote landscapes is a notoriously costly and difficult task. The emergence of small-Unmanned Aircraft Systems (sUAS) equipped with low-cost multi-spectral cameras offer an alternative to traditional grassland survey methods, and have the potential to progress and innovate the monitoring and future conservation of this habitat globally. The aim of this article is to investigate the potential of sUAS for rapid detection of threatened unimproved grassland and to test the use of an Enhanced Normalized Difference Vegetation Index (ENDVI). A sUAS aerial survey is undertaken at a site nationally recognised as an important location for fragmented unimproved mesotrophic grassland, within the south east of England, UK. A multispectral camera is used to capture imagery in the visible and near-infrared spectrums, and the ENDVI calculated and its discrimination performance compared to a range of more traditional vegetation indices. In order to validate the results of analysis, ground quadrat surveys were carried out to determine the grassland communities present. Quadrat surveys identified three community types within the site; unimproved grassland, improved grassland and rush pasture. All six vegetation indices tested were able to distinguish between the broad habitat types of grassland and rush pasture; whilst only three could differentiate vegetation at a community level. The Enhanced Normalized Difference Vegetation Index (ENDVI) was the most effective index when differentiating grasslands at the community level. The mechanisms behind the improved performance of the ENDVI are discussed and recommendations are made for areas of future research and study.

## 1. Introduction

The recent emergence and availability of small-Unmanned Aircraft Systems (sUAS) presents new possibilities for landscape scale ecological assessment. This rapidly developing field has been shown, in some cases, to be more effective than more traditional remote sensing methods in meeting the requirements of researchers seeking fast, adaptable and successful monitoring of management initiatives and approaches [1–2]. High costs, low resolution, and the lack of flexibility often associated with satellites and piloted aircraft have limited their widespread application [3]. Thus, sUAS present new approaches to aerial research and remote sensing as they are lightweight, relatively low-cost, and capable of carrying a developing range of sensors and imaging equipment [4].

The repeatable and cost-effective nature of sUAS methods means that these systems can be adapted to operate at a range of appropriate spatial and temporal scales. Furthermore, the replicable nature of sUAS surveys facilitates assessment of rapid temporal changes in land cover and habitats [4], and potentially for seasonal changes to be recorded and monitored. The development of improved sensors and innovations in spectral indices (e.g. narrow band, red-edge) is furthering their application and offering the opportunity to discern landscape differences at smaller resolutions and finer spatial and temporal scales [5–7].

Unmanned aircraft systems have found application in a broad range of environmental research fields. Examples include large mammal population surveys [8–9], the monitoring of breeding bird colonies [10] and precision agriculture [11–14]. However, sUAS also have the potential to transform the way in which semi-natural vegetation surveys are conducted [7, 15–16]. The ability of this technology to fly at low altitudes and be equipped with appropriate sensors enables the detection of species assemblages [7] and spatial variations in plant community structure to be observed [16].

One area in which sUAS may be of particular use is the surveying of, amongst others, semi-natural and unimproved grassland habitats. The widespread loss and degradation of unimproved grassland worldwide, particularly across Europe, means that these habitats are of increasing conservation concern [17–20]. Locating and monitoring remnant patches of this habitat is critically important to ensure that they are afforded protection and not lost completely [21–23]. However, traditional surveying of large, remote landscapes is a notoriously difficult task. In addition to time costs associated with large areas and the need for repeat surveys [24], further issues relating to site access mean that surveying is challenging and potentially costly [25]. The development of a semi-automated method has the potential to significantly change the way in which internationally important semi-natural grasslands are surveyed and monitored. The development of a rapid sUAS methodological approach, along with the use of well-understood vegetation indices, could facilitate widespread assessment of temporal changes, habitat loss, and the effectiveness of management initiatives [4].

This paper presents a novel approach for rapid surveying of semi-natural grassland systems using a low cost sUAS. A semi-automated method of detecting unimproved grassland habitat at the community level is presented, and the effectiveness of an Enhanced Normalised Vegetation Difference Index compared to a range of traditional vegetation indices.

The research objectives were to: 1) acquire aerial imagery of the study site in both standard visible (RGB) and near-infrared (NIR) spectrums using a sUAS; 2) Undertake quadrat surveys of vegetation communities present at the study site in order to ground-truth accuracy and effectiveness of a sUAS approach; 3) Calculate and compare the effectiveness of a range of vegetation indices to differentiate between improved and unimproved grassland communities.

## 2. Vegetation indices

Vegetated habitats can be remotely sensed through the calculation of vegetation indices from satellite or aerial imagery. First developed by Rouse *et al*. [26] in 1974, The Normalized Difference Vegetation Index (NDVI) is the most prominently used of vegetation indices [27–32]. NDVI is largely used to detect live vegetation by measuring the reflectance levels of visible red and near-infrared light [30]. The NDVI is based on the principle that healthy vegetation absorbs a large proportion of the visible light that reaches it whilst reflecting most of the radiation in the near-infrared region [30]. Plant cells have evolved to reflect radiation in the near-infrared spectrum (700–1400 nm) as the larger wavelengths found in this region do not create sufficient photon energy to produce organic molecules [33]. NDVI has been used in a broad range of applications including the estimation of crop yield [29, 32], measuring deforestation rates [28], and following drought [31]. NDVI has also been used within ecological research to identify the extent of natural habitats and examine spatial and temporal changes [27].

Since the first research using NDVI, a number of alternative vegetation indices have been developed. The majority of these are adapted from NDVI and combine two or more spectral bands. A series of those deemed important to vegetation studies are shown in Table 1. Following the initial use of the red band to calculate NDVI, attention has moved towards the application of the green and blue wavelengths. In a study of tree canopy variation, Gitelson *et al*. [34] showed that the green band displayed a greater sensitivity to chlorophyll concentrations than the red channel. The report also demonstrated that the use of green wavelengths resulted in more accurate measurements of pigment concentrations and led to the development of the Green Normalized Difference Vegetation Index (GNDVI) [34].

**Table 1:** Examples of vegetation indices developed for remote sensing applications.

Vegetation Index	Equation	Reference
Normalized Difference Vegetation Index (NDVI)	(NIR—R) / (NIR + R)	Rouse *et al*., 1974 [26]
Green Difference Vegetation Index (GDVI)	NIR—G	Sripada *et al*. 2006 [36]
Green Normalized Difference Vegetation Index (GNDVI)	(NIR—G)/(NIR + G)	Gitelson *et al*. 1996 [34]
Enhanced Normalized Difference Vegetation Index (ENDVI)	((NIR + Green)—(2*Blue) / ((NIR + Green) + 2*Blue))	Maxmax, 2015 [39]
Green Infrared Percentage Vegetation Index (GIPVI)	NIR / (NIR + Green)	Crippen, 1990 [38]
Green Ratio Vegetation Index (GRVI)	NIR / Green	Sripada *et al*. 2006 [36]

The Difference Vegetation Index (DVI), first developed by Tucker [35] in 1979, is calculated by subtracting reflectance from the red channel by that in the NIR [35]. Sripada *et al*. [36] modified the DVI by substituting the red band for the green to form the Green Difference Vegetation Index (GDVI). The GDVI was utilised to determine in-season nitrogen requirements for corn crops. Another vegetation index developed by Sripada *et al*. [36] is the Green Ratio Vegetation Index (GRVI), which was modified from an original Ratio Vegetation Index (RVI) established by Birth and McVey [37] in 1968. Utilisation of the green band means that the GRVI is less sensitive to variations in ground cover as vegetation has a higher level of reflectance for green wavelengths [36].

The red channel is also substituted for the green band in the GIPVI, a variation of the Infrared Percentage Vegetation Index (IPVI) developed by Crippen [38] in 1990. Calculated by dividing the NIR band by the sum of the NIR red bands combined, the IPVI measures the percentage of NIR radiance relative to the combined radiance of both bands [38].

The Enhanced Normalized Difference Vegetation Index (ENDVI) developed by LDP LLC, Carlstadt, NJ, USA [39] incorporates three spectral bands (NIR, B, and G) to produce better discrimination within the index in comparison to the original NDVI. By using both the NIR and green channels, the index inflates the chlorophyll reflection values by summing both NIR reflectance and green channel reflectance [39]. Furthermore, the inclusion of the blue channel in the index has the potential to amplify the recorded chlorophyll absorption values due to the increased amplitude of absorption of blue wavelength energy, particularly chlorophyll-*b*, in this part of the electromagnetic spectrum. The ENDVI has recently found application within agricultural monitoring [13–14], measuring peatland disturbance [40] and orchard management [12].

With the recent emergence of sUAS, there is an opportunity to advance upon the application and conceptualisation of indices traditionally calculated using imagery obtained from satellites and manned aircraft. SUAS present a platform for new possibilities for rapid and adaptive spectral analysis of vegetation.

## 3. Methods

### 3.1 Study site

**Fig 1:**
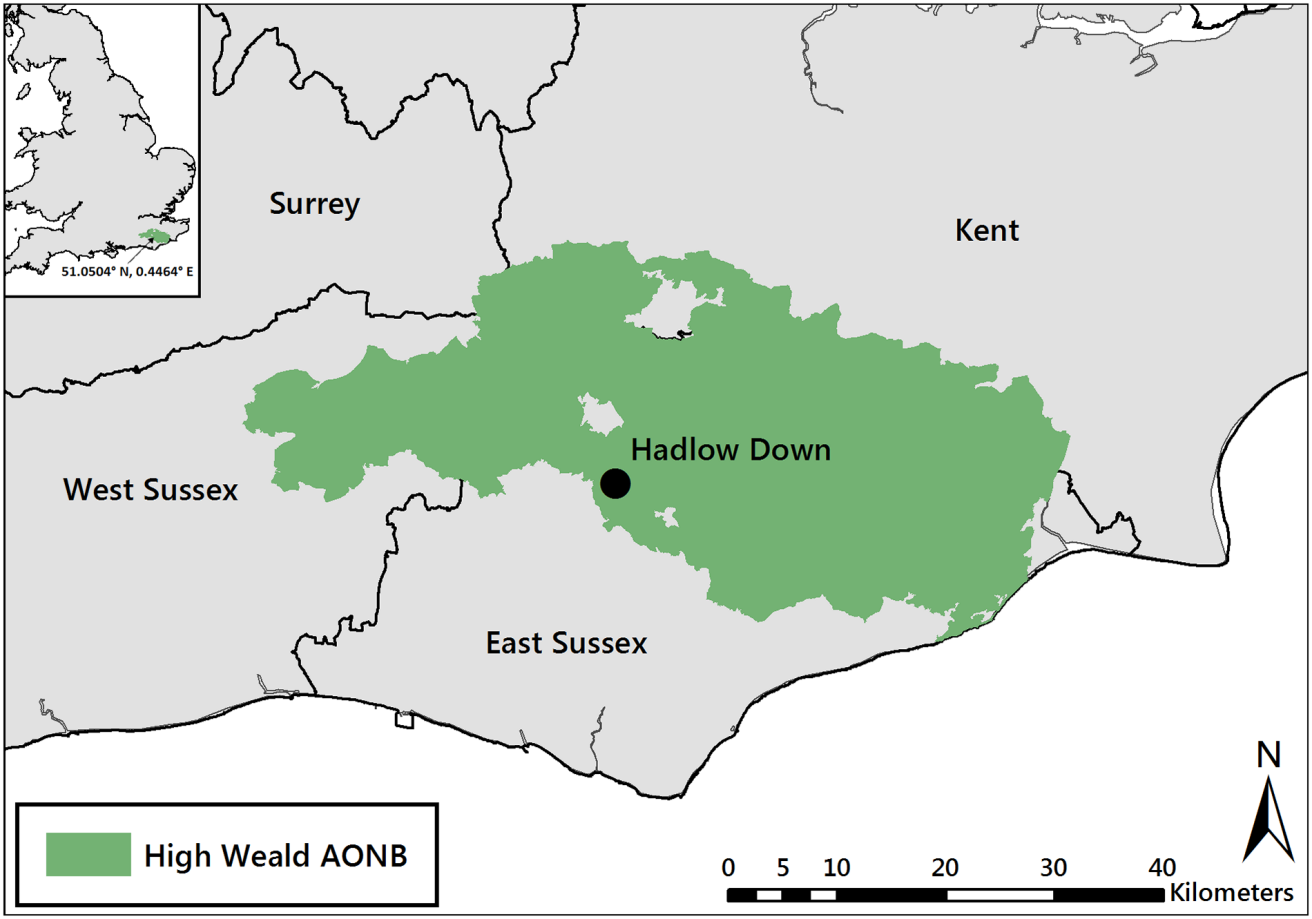
The location of the study site within the High Weald AONB in south east England. Reprinted from Ordnance Survey (Digimap Licence) under a CC BY license, with permission from Crown Copyright and Database Right [2017].

The study was undertaken within the High Weald Area of Outstanding Natural Beauty (AONB). The High Weald is located in south east England (East Sussex, West Sussex, Kent and Surrey) and was recognised nationally as an AONB in 1983. The landscape covers an area of 1461 km^2^ (Fig 1), and exists as a mosaic of different habitats including woodland, hedgerows, heathland, scattered farmsteads and grassland [41]. There are approximately 305, heavily fragmented, unimproved grassland sites scattered across the AONB representing a total area of 6.6 km^2^. Many of these are small in size, with the largest being only 0.3 km^2^ [25].

**Fig 2:**
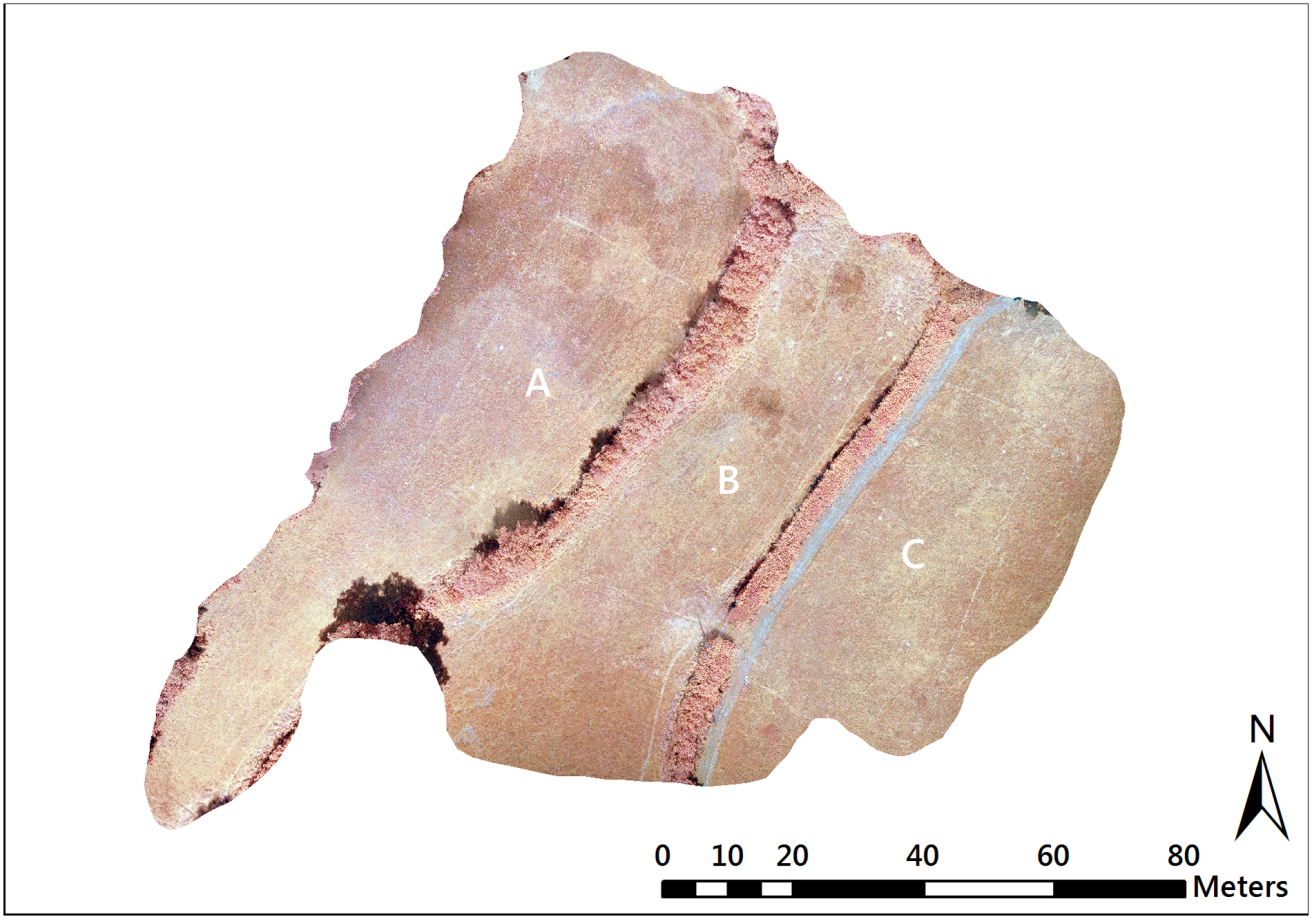
Near Infra-Red (NIR) aerial image of study site target analysis area located at Upper Spoods Farm, Hadlow Down, East Sussex. Fields A and B = MG5c unimproved grassland, field C = MG6b improved grassland. The white line represents the small brook that runs along the hedgerow separating fields A and B.

The survey location was Upper Spoods Farm in Hadlow Down (NGR TQ538234; 85 m ASL, Fig 1), in an open farmland/forested area. High Weald AONB Authority and the landowners of Upper Spoods Farm provided site permission. The total survey site covered an area of 4,164m^2^ and was comprised of three grassland fields (fields A, B, and C) separated by mixed species hedgerows (Fig 2). An area of deciduous woodland bordered the site to the north and east whilst the remaining area adjacent to the site was either continuation of grassland habitats, or farmland intersected by hedgerows. The area had previously been identified as a potential survey site by the High Weald AONB unit, due to the presence of nationally important unimproved mesotrophic grassland communities.

The eastern field (C) was predominantly improved MG6b (*Lolium perenne-Cynosurus cristatus*) communities whilst the central (B) and western field (A) consisted of unimproved MG5c (*Cynosurus cristatus-Centaurea nigra*) communities. Discernible patches of M23 (*Juncus effusus/acutiflorus-Galium palustre*) rush pasture also existed in fields A and B, lying close to the small brook that dissects the site. As the site was bounded by dense woodland, it was also ideal for testing the adaptive and nimble nature of sUAS approaches for landscape assessment. Furthermore, the range of habitat types present within a small area made the site suitable as it allowed assessment of the effectiveness of a multispectral sensor to distinguish between the habitats and communities present at the small scale.

### 3.2 Vegetation quadrat surveys

Vegetation quadrat surveys were undertaken over two days during July 2016. Species presence within 1m^2^ quadrats were recorded and assigned a Domin scale value of percentage cover. The results of the quadrat surveys were then used to assign each of the three fields in a hierarchical manner to a National Vegetation Classification (NVC) community type [42]. A total of twenty-nine quadrats were randomly sampled across the study site and represented all predominant grassland habitat types present. GPS locations were recorded for quadrats that were orientated northwards. Quadrats were surveyed in a consistent manner and both presence/absence and percentage cover were recorded. One-way ANOVA (analysis of variance) tests were performed (Minitab v.17) in order to determine the existence of significant differences between mean species numbers and habitat composition. Following ANOVA, post-hoc analysis using Tukey's HSD (Honest Significant Difference) test identified significant differences between pairs of habitat types.

### 3.3 Image acquisition

Aerial imagery was acquired using a DJI Inspire 1 sUAS. The sUAS was set to a target altitude of 25m above ground level (AGL) and was flown at 5 meter line spacing in consistent weather conditions (temperature:18°C, wind speed: 3.6ms^-1^ NW, sun with minor cloud cover). Following an initial test flight, aerial images were acquired during four separate flights over an hour-long period. These followed a crosshatched flight plan to ensure maximum overlap (>80%) and complete coverage of the site. Images were captured using both a standard RGB 12 megapixel DJI Zenmuse X3 camera (DJI, Europe) and a modified BG-NIR version of this sensor. The modified camera contained a custom filter that passes infrared light from the ‘red edge’ at 680-800nm where plants actively reflect wavelengths. By blocking wavelengths over 800nm, the filter ensures that the blue and green channels only receive visible light whilst allowing the detection of NIR light at 680-800nm (LDP LLC, Carlstadt, NJ, USA).

A total of eighteen ground control points (GCPs) were used during the aerial field survey. The GCPs were surveyed using a Leica GPS1200 differential global positioning system (dGPS) and were post-processed using Leica Geo Office. The raw GPS data collected using the dGPS required post processing using RINEX (Receiver Independent Exchange Format) data. The sUAS was set to acquire images at timed intervals every 5 seconds with 356 images captured. All images acquired during the field survey were recorded in a JPEG file format and georeferenced to EXIF GPS coordinates and altitude level obtained from the DJI Inspire 1 sUAS.

### 3.4 Image processing and analysis

Of the original 356 images captured, 351 were selected for processing using the Agisoft Photoscan v1.2.5 (build 2735) software product. An estimate of image quality was used to assess the suitability of images for their inclusion in processing. Images were downscaled and then compared to the originals, giving each image a value based on sharpness. Images with a value greater than 0.70 were included in image processing. Orthorectified images of the study site were produced from both the visible spectrum and NIR imagery. A Structure-from-Motion (SfM) Digital Surface Model (DSM) was created for ortho-rectification purposes [43]. Image processing followed the standard Agisoft procedure [44].

Nine of the eighteen coded Ground Control points (GCPs) were used to optimise camera position and orientation. A dense point cloud was produced from the estimated camera locations, and aggressive depth filtering used to remove outliers. The generated DSM was then used to produce orthorectified images for the study site.

Additional image analysis was undertaken using ArcMap v10.3.1 (ESRI, 2015). Orthorectified imagery was separated into four layers based on spectral bands (red, green, blue, NIR). Images were clipped to the study site boundary and hedgerows dividing each field were removed, allowing greater zonation within the grassland fields. Vegetation indices (Table 1) were calculated to test for differences in the target fields. By determining the average index values for quadrat locations within the target fields, a spectral signature file was generated for future application. A total of six vegetation indices were calculated (Table 1).

Extraction method was used to determine mean index values for all six indices in each of the quadrats. Statistical analysis was undertaken to determine existence of significant differences between habitat and community types within each field. One-way ANOVA tests with post-hoc Tukey comparisons were performed. Where normality assumptions were not met, Kruskal-Wallis tests were undertaken. Differences between broad habitats (e.g. rush and grassland) were initially examined, with findings subsequently used to analyse differences between NVC communities and sub-communities.

Finally, for independent assessment of the sUAS image accuracy, the remaining nine GCPs were recorded by dGPS and compared to the marked locations on the orthophotos. Root Mean Square Error (RMSE) and Mean Absolute Error (MAE) were calculated to estimate differences between image-based control points and independent dGPS data.

## 4. Results

### 4.1 Image processing output

The dense point cloud produced from the imagery was comprised of 11,406,037 matched points. The effective overlap of photographs was less than nine images per point within the study site. The DSM had a reported resolution of 0.0384m per pixel, and the resolution of the orthorectified image was 0.0096m per pixel. Photoscan (Agisoft, 2016) reported total RMSE values of 0.0382m for the orthophoto.

### 4.2 Model accuracy

Independent accuracy assessment of the orthomosaic and DSM output was conducted. Independent ground control points, obtained using a Leica dGPS, were compared to concurrent locations on the orthomosaic image and DSM. RMSE and MAE values for the nine independent ground control points showed that the positional accuracy of the orthomosaic was between 0.048m RMSE (x-axis) and 0.054m RMSE (y-axis), and elevational accuracy of the model was 0.335m RMSE (z-axis) (Table 2).

**Table 2:** Comparison of combined XYZ coordinates between DSM and independent dGPS ground control points with Root Mean Square Error (RMSE) and Mean Absolute Error (MAE) values.

	Difference from dGPS points (m)		
Control Pt	x axis	y axis	z axis
**1**	-0.049	0.021	0.079
**2**	-0.014	0.006	0.196
**3**	-0.017	-0.022	0.047
**4**	0.002	-0.009	0.239
**5**	-0.018	0.035	0.287
**6**	0.028	-0.062	0.119
**7**	0.129	-0.114	-0.867
**8**	0.001	-0.047	-0.234
**9**	-0.009	-0.070	0.067
**RMSE (all pts)**	0.048	0.054	0.335
**MAE (all pts)**	0.030	0.043	0.237

### 4.3 Vegetation survey results

Forty-one plant species were identified within the study site and this information was used to assign each of the three fields the following NVC classifications:

- **MG5c**: (*Cynosurus cristatus-Centaurea nigra* grassland, *Danthonia decumbens* subcommunity): **Fields A and B**.
- **MG6b**: (*Lolium perenne-Cynosurus cristatus* grassland): **Field C**.
- **M23**: (*Juncus effusus*/*acutiflorus—Galium palustre* rush pasture): **Individual rush patches in fields A and B**.

**Fig 3:**
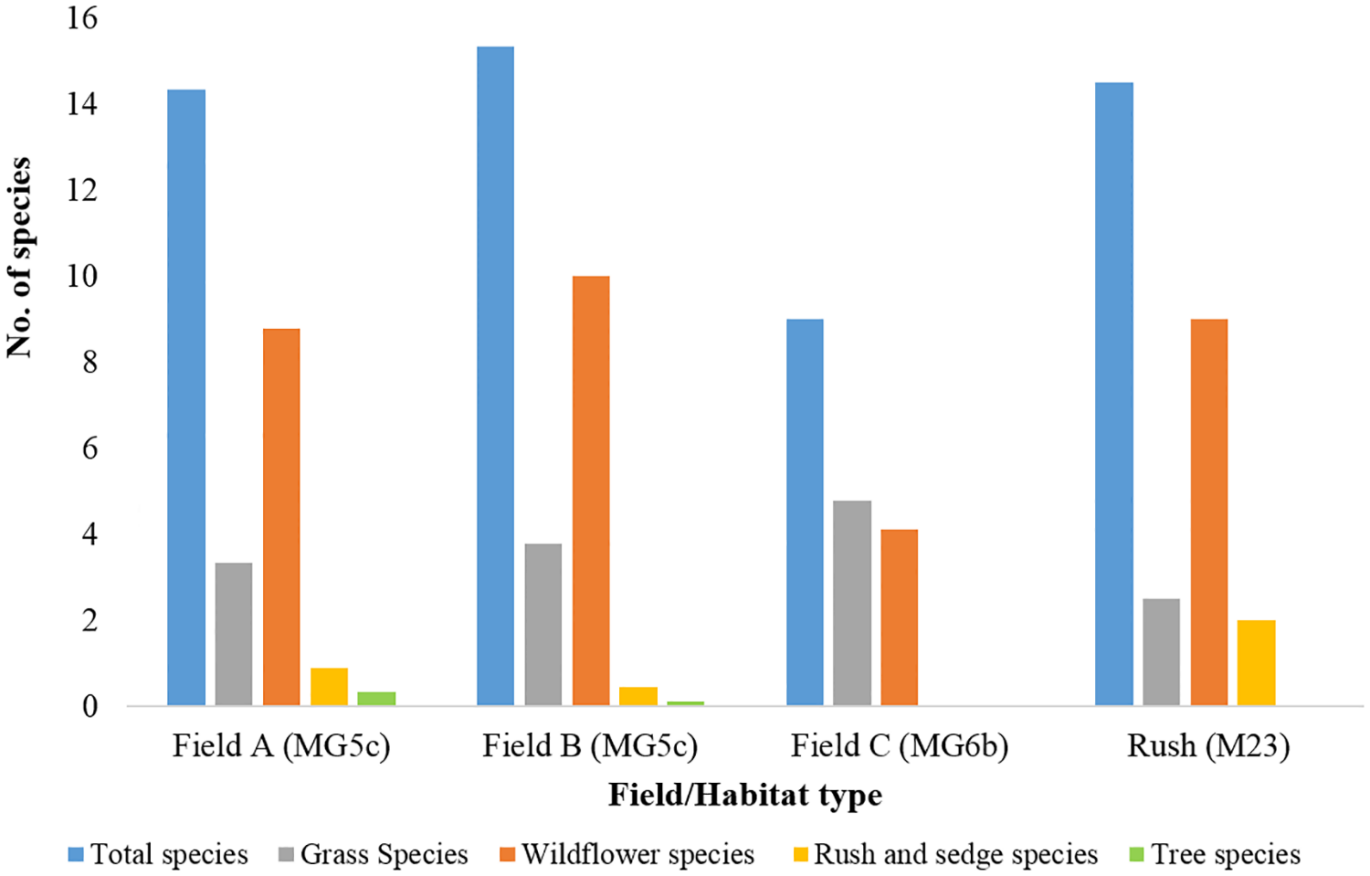
Mean number of total species and species by group for quadrats within each field/habitat type.

Fig 3 shows the average number of total species, as well as species by group, for each field/habitat type.

The quadrat surveys showed that unimproved grassland (MG5c) was found to have a higher mean total number of plant species (14.3 and 15.3) than improved grassland (9.0). The total number of species within the two rush patches (M23) was also higher, with an average of 14.5 species per quadrat. Improved grassland also had a lower mean number of wildflower species (4.2) compared to the two unimproved fields (9.8 and 11.0). In contrast, grass species were less abundant within unimproved fields (3.3 and 3.8) than improved grassland (4.8). The two rush patches had the highest mean number of rush and sedge species (2.0) in comparison to unimproved grassland (0.9 and 0.4) and the improved field in which they were absent. Finally, a small number of tree saplings were found in fields A and B at the western edge of the study site and were a result of encroachment from the adjacent woodland.

The results of one-way ANOVA tests (Table 3) showed that four of the five species groups differed significantly between habitat types at the 95% confidence level. The average number of tree species was the only group that did not vary between habitats due to a lack of tree specimens (F = 0.86, P = 0.4358).

**Table 3:** One-way ANOVA results for species groups and Tukey HSD post-hoc comparisons.

	ANOVA	Tukey test for difference of means		
		Comparison groups	Mean difference	Post-hoc P-value
**Total species**	F = 13.25.0001*	MG5c-MG6b	-4.83	0.0010*
	P = 0.0001*	MG5c-Rush	0.67	0.9000
		MG6b-Rush	5.50	0.0478*
**Grass species**	F = 10.205*	MG5c-MG6b	1.22	0.0024*
	P = 0.0005*	MG5c-Rush	-1.06	0.1968
		MG6b-Rush	-2.28	0.0031*
**Wildflower species**	F = 26.19	MG5c-MG6b	-6.17	0.0010*
	P = <0.0001*	MG5c-Rush	-0.39	0.9000
		MG6b-Rush	5.78	0.0046*
**Rush and sedge species**	F = 5.43	MG5c-MG6b	-0.67	0.1529
	P = 0.0107*	MG5c-Rush	1.33	0.0887
		MG6b-Rush	2.00	0.0115*
**Tree (saplings) species**	F = 0.86	-	-	-
	P = 0.4358	-	-	-

Note:

* indicates a statistically significant difference with p< 0.05.

Post-hoc comparisons using the Tukey HSD test revealed that improved grassland (MG6b) contained a significantly lower mean number of total species than unimproved grassland (MG5c) (P = 0.0010) with the latter possessing the higher values. Rush pasture (M23) was found to have a significantly different number of total species in comparison to improved grassland (P = 0.0478) with the former possessing the higher values, but no significant difference was observed between rush and unimproved grassland (P = 0.9000).

Comparisons for the mean number of grass species identified differences between two groups. Quadrats within the improved grassland were found to have a significantly different number of grasses than those in unimproved grassland (P = 0.0024) and the rush patches (P = 0.0031) with the improved grassland consistently showing the higher values. For wildflower species, improved grassland was found to have a significantly different number of species than both unimproved grassland (P = 0.0010) and rush patches (P = 0.0046) with the improved grassland consistently showing the lower values on this occasion. Rush patches were found to have a significantly different number of rush and sedge species in comparison to improved grassland (P = 0.0115), but not for unimproved grassland (P = 0.0887).

### 4.4 Vegetation indices

**Fig 4:**
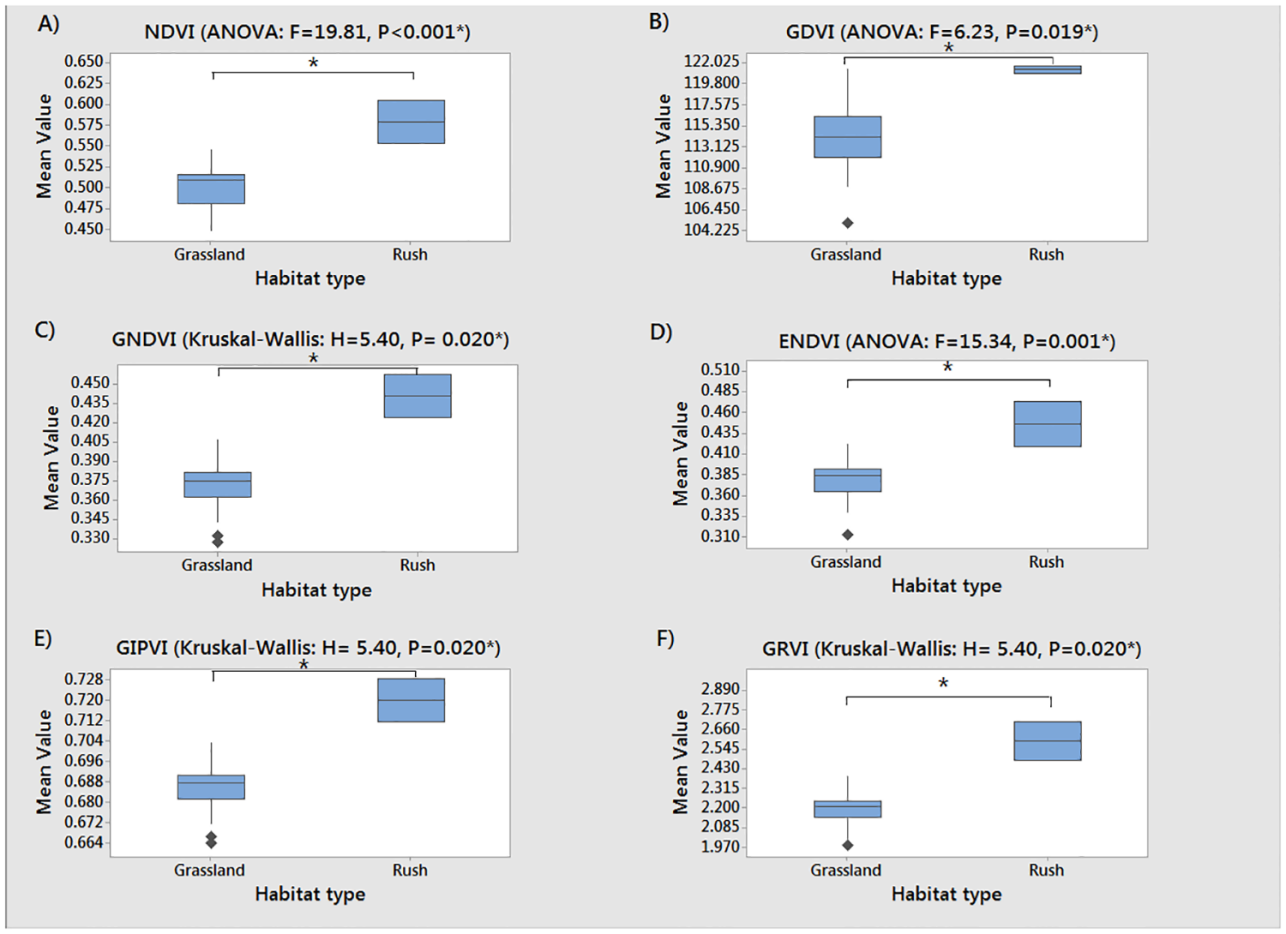
Mean vegetation index values for 1m^2^ quadrats within grassland and rush habitats. A = Normalized Difference Vegetation Index (NDVI), B = Green Difference Vegetation Index (GDVI), C = Green Normalized Difference Vegetation Index (GNDVI), D = Enhanced Normalized Difference Vegetation Index (ENDVI), E = Green Infrared Percentage Vegetation Index (GIPVI), F = Green Ratio Vegetation Index (GRVI).

The boxplots displayed in Fig 4 show the vegetation indices calculated from the sUAS captured imagery at the habitat level (grassland vs rush habitat). ANOVA and Kruskal-Wallis tests identified significant differences between the two main habitat types for all indices. Additionally, the mean index value of the rush habitat was visibly higher than that of grassland in all six cases.

**Fig 5:**
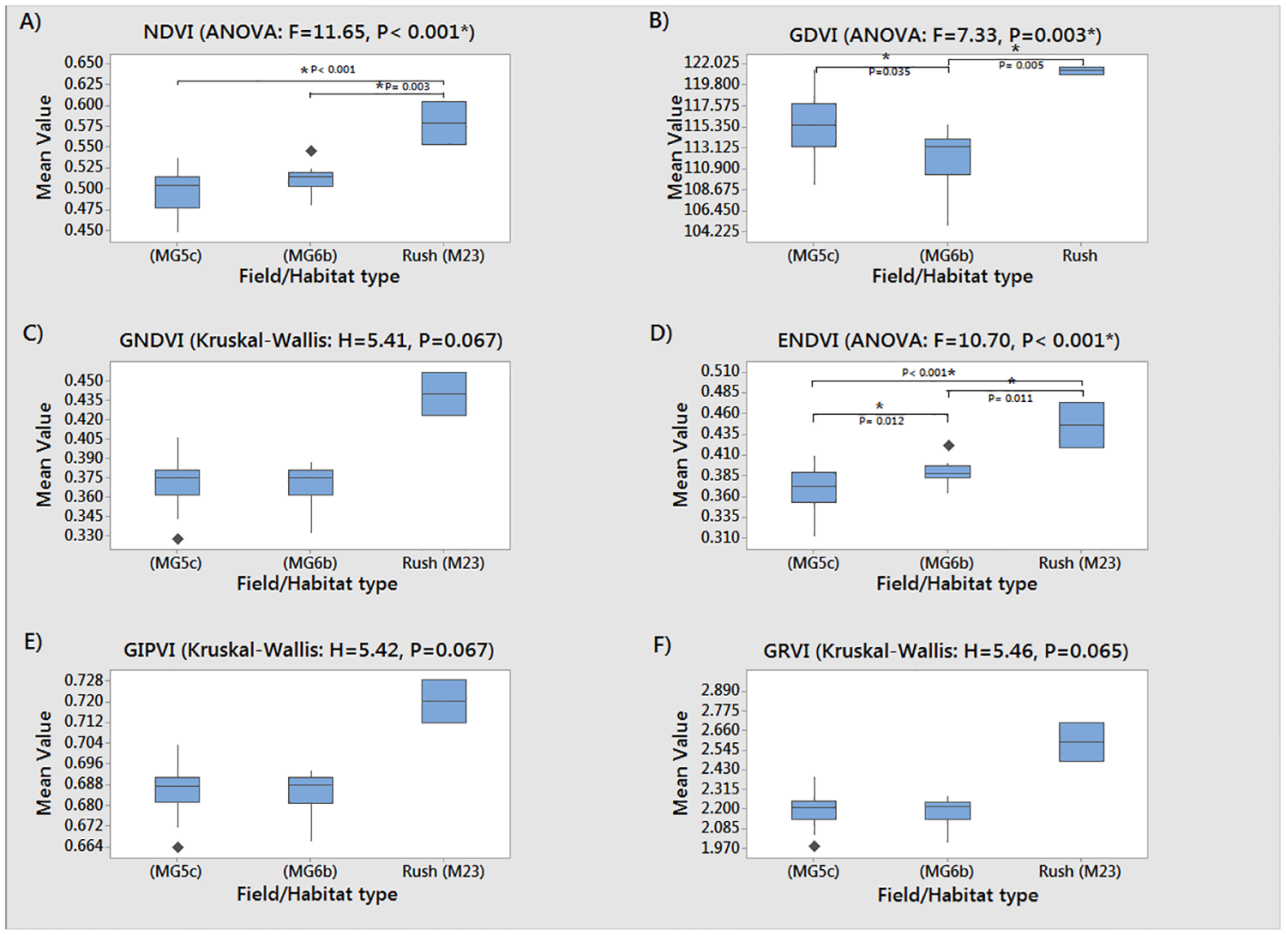
Mean vegetation index values for 1m^2^ quadrats within each of the community types. MG5c = Unimproved grassland, MG6b = Improved grassland. A = Normalized Difference Vegetation Index (NDVI), B = Green Difference Vegetation Index (GDVI), C = Green Normalized Difference Vegetation Index (GNDVI), D = Enhanced Normalized Difference Vegetation Index (ENDVI), E = Green Infrared Percentage Vegetation Index (GIPVI), F = Green Ratio Vegetation Index (GRVI). * represent significant differences at <0.05 significance between habitat types detected using Tukey post-hoc analysis.

Following habitat level analysis, boxplots were calculated at the community level (Fig 5) to identify differences in the vegetation indices values between the three NVC categories present within the site (MG5c and MG6b grassland, M23 rush pasture). Again, the mean index value of the rush habitat was visibly higher than that of grassland in all cases and significant differences between the groups were detected to at least the 93% confidence level for all examples.

The boxplots displayed in Fig 5 show the vegetation indices calculated at the community level (MG5a, MG6b, and M23). Significant differences above the 95% confidence level between community types were identified in only three of the six indices (NDVI: F = 11.65, P< 0.001, GDVI: F = 7.33, P = 0.003, and ENDVI: F = 10.70, P< 0.001). Post-hoc Tukey comparisons showed that all three indices were able to display a significantly different mean index value between (at least one of) the grassland and rush pasture communities. Both ENDVI (p = 0.012) and GDVI (p = 0.035) also showed a significant difference between unimproved (MG5c) and improved (MG6b) grassland communities, but the latter was unable to discern significant differences between improved (MG6b) and rush-pasture (M23), indicating that GDVI may not be suitable for this purpose.

Statistical analysis determined that ENDVI was the most effective in representing and identifying the differences between all NVC community types studied at the quadrat level. ENDVI was the only index capable of showing significant difference in spectral response data across all NVC community combinations (MG5c unimproved grassland, MG6b improved grassland, and M23 pasture-rush).

## 5. Discussion

### 5.1 DSM and orthophoto accuracy

The assessment of GCP locations found that the positional error for the nine independent control points was small (0.048–0.054m), and suggested that the DSM and orthophoto outputs were a strong representation of positional field measurements. The elevation error was larger (0.335m) than positional values, but can be explained by the structure of the grassland vegetation within the site. During quadrat surveys, the mean vegetation height was measured at 0.208m. This factor is likely to have resulted in the vertical difference observed between sUAS-derived DSM elevation values and those obtained using the dGPS at ground level. These findings are similar to those observed by Tonkin *et al*. [45] in two contrasting mountainous areas. In their study, the value for a less densely vegetated area (0.200m) was found to be significantly lower than for an area of high vegetation cover (0.588m), corresponding to the vertical difference observed during this study [45].

### 5.2 Vegetation indices

Initial vegetation quadrat surveys identified two clear habitat types within the study site; mesotrophic grassland (MG) and rush pasture (M). Further analysis at the community level found fields A and B to be comprised of unimproved MG5c (*Cynosurus cristatus-Centaurea nigra*) grassland whilst field C contained improved MG6b (*Lolium perenne-Cynosurus cristatus*) habitat. Scattered patches of distinctive rush pasture (M23) were evident in fields A and B. By determining the species assemblages present at the study site, and recording sample quadrat locations, vegetation indices were calculated from aerial imagery to determine if these differences were discernible remotely.

All six of the vegetation indices recorded from a sUAS, using a multispectral camera, were able to distinguish between MG mesotrophic grassland and M23 rush pasture at the habitat level. The mean index values for rush pasture were higher than that of grassland in all cases (Fig 4). Index values showed that rush patches presented comparatively lower NIR+green reflectance values and lower reflectance of the blue wavelength energy. Conversely, grassland areas presented higher values of NIR+green reflectance, but lower reflectance of the blue wavelength energy. Vegetation surveys of the rush patch quadrats revealed that, as would be expected, this habitat type had the highest mean number of rush and sedge species present (2.0). The rush patches were also characterised by having the lowest mean number of grass species (2.5) of the three habitat types. Results of the quadrat surveys showed that these patches were dominated by sharp-flowered rush (*Juncus acutiflorus*), and to a lesser extent, compact rush (*Juncus conglomeratus*). During field surveys it was evident that this habitat was visually different to the surrounding grassland, with dark green rush plants forming dense clumps. There have been a number of previous studies that have highlighted the high vegetation index values associated with *Juncus* rush habitats [46–47], reflecting the results observed in this report.

As analysis progressed beyond simple habitat assessment, to community level investigation, only three of the six vegetation indices (NDVI, GDVI, ENDVI) were able to identify specific differences between the NVC communities present at the study site (MG5c unimproved grassland, MG6b improved grassland, and M23 rush pasture) above the 95% threshold confidence level. The same general patterns of reflectance were evident at the community level with higher index values produced from the rush pasture than those of the grassland communities.

The ENDVI was the only index that was able to separate all combinations of communities. The ENDVI separated the rush pasture (M23) and both grassland communities (MG5c: P<0.001; MG6b: P = 0.011); whilst contemporaneously distinguishing between unimproved (MG5c) and improved (MG6b) grassland communities (P = 0.012). MG5c communities were consistently shown to present lower index values than the improved MG6b communities. In terms of the nature of the ENDVI index, the spectral reflectance data showed that both communities presented similar ‘difference values’ between the NIR+green and blue bands. However, the comparative increase in the ‘total amount of energy’ reflected by unimproved grassland across the three available bands enabled the two communities to be separated within the imagery. The distinction in the reflectance properties of these two communities may, in part, be a result of the significant differences present in the abundance of wildflower species (MG5c = 10.5, MG6b = 4.2) and the higher abundance of grass species (mean species number: MG5c = 3.6, MG6b = 4.8).

The unimproved (MG5c) grassland in field A had the lowest mean number of grass species (3.3 per quadrat). This increased marginally in field B (3.8 per quadrat), and more substantially in the improved grassland field C (MG6b; 4.8 per quadrat). The dominance of grasses and lack of wildflower species (4.2) within the improved field C directly contrast to the unimproved fields in which fewer grass species were present and the mean number of wildflowers were considerably higher (9.9 and 11.1). The differing spectral properties associated with these two vegetative groups may explain why vegetation index values were different between fields and community types. It is likely that the reason the ENDVI was the most effective index at distinguishing between grassland communities is due to it incorporating three spectral bands (blue, green and NIR).

This ENDVI index was developed by LDP LLC, Carlstadt, NJ, USA [39] and is proposed as an improvement to the NDVI in these low flight-height sUAS surveys of grassland communities. It differs from the other indices tested by incorporating the green, blue and NIR spectral bands [39]. As stated previously, by summing the NIR and green channels together, the ENDVI amplifies the chlorophyll reflection of vegetation imagery [12]. More specifically, the inclusion of the green band is suggested to increase sensitivity to chlorophyll concentrations [34]. The further inclusion of the blue channel provides a bigger potential response range resulting from chlorophyll *b*; increasing the dynamic range of the resultant index values. The carotenoid concentrations in the grassland species may be another key group, absorbing violet and blue-green light and functioning as light capture and photo protective pigments. It is possible that these factors, in particular the increased amplitude of absorption of blue wavelength energy by chlorophyll *b* (453nm maximally), and carotenoids (400-500nm maximally) enabled the ENDVI to provide better discrimination. During this project, the ENDVI was found to be more effective at distinguishing between improved and unimproved grassland communities, although further study is required.

The ENDVI has found application in agricultural monitoring [13–14], measuring peatland disturbance [40] and the management of orchards [12]. As far as known, this project is the first to apply the ENDVI for the identification of grassland habitats. This project has shown the effective ENDVI indices calculated from sUAS-acquired imagery can be used to distinguish grassland vegetation to a community level. However, further research is needed to refine the way in which aerial imagery is analysed so that grassland habitats can be more effectively separated. Future studies within this field should build upon the results of this study, in particular the potential of the ENDVI for distinguishing between grassland habitats and the identification of unimproved fragments. Further research by the authors will focus on investigating the ability of the ENDVI to identify plant communities and species with known variations of Chlorophyll *a–b* and Carotenoid content and seasonal/stress variation. Future research should also focus on integration and utilisation of narrow-band and ‘red-edge’ spectral band (680-740mm) to differentiate between habitats, communities and species. The ‘red-edge’ band exists within the transition zone between the red and NIR regions, representing the boundary between chlorophyll absorption and scattering by internal leaf structure [48]. Vegetation indices that incorporate the red-edge band have been shown to more accurately estimate green leaf area index (LAI) than the traditional NDVI [5]. These indices have the potential to distinguish between similar vegetation communities and their effectiveness for this application should be explored in future research.

## Supporting information

S1 Fig Visible spectrum (RGB) aerial image of study site target analysis area located at Upper Spoods Farm, Hadlow Down, East Sussex. Fields A and B = MG5c unimproved grassland, field C = MG6b improved grassland.
(TIF)

S1 Table A summary of mean species values, standard deviation (S.D.) and 95% confidence intervals (CI) for the vegetation survey data. (DOCX)

S2 Table Vegetation index statistics (Min value, max value, mean, standard deviation) for each of the three habitat communities present within the study site. (DOCX)
