# Supplementary figures and images for: The potential of small-Unmanned Aircraft Systems for the rapid detection of threatened unimproved grassland communities using an Enhanced Normalized Difference Vegetation Index
Source: PLoS One. 2017 Oct 12;12(10):e0186193. doi: 10.1371/journal.pone.0186193 (PMC5638390; doi:10.1371/journal.pone.0186193)

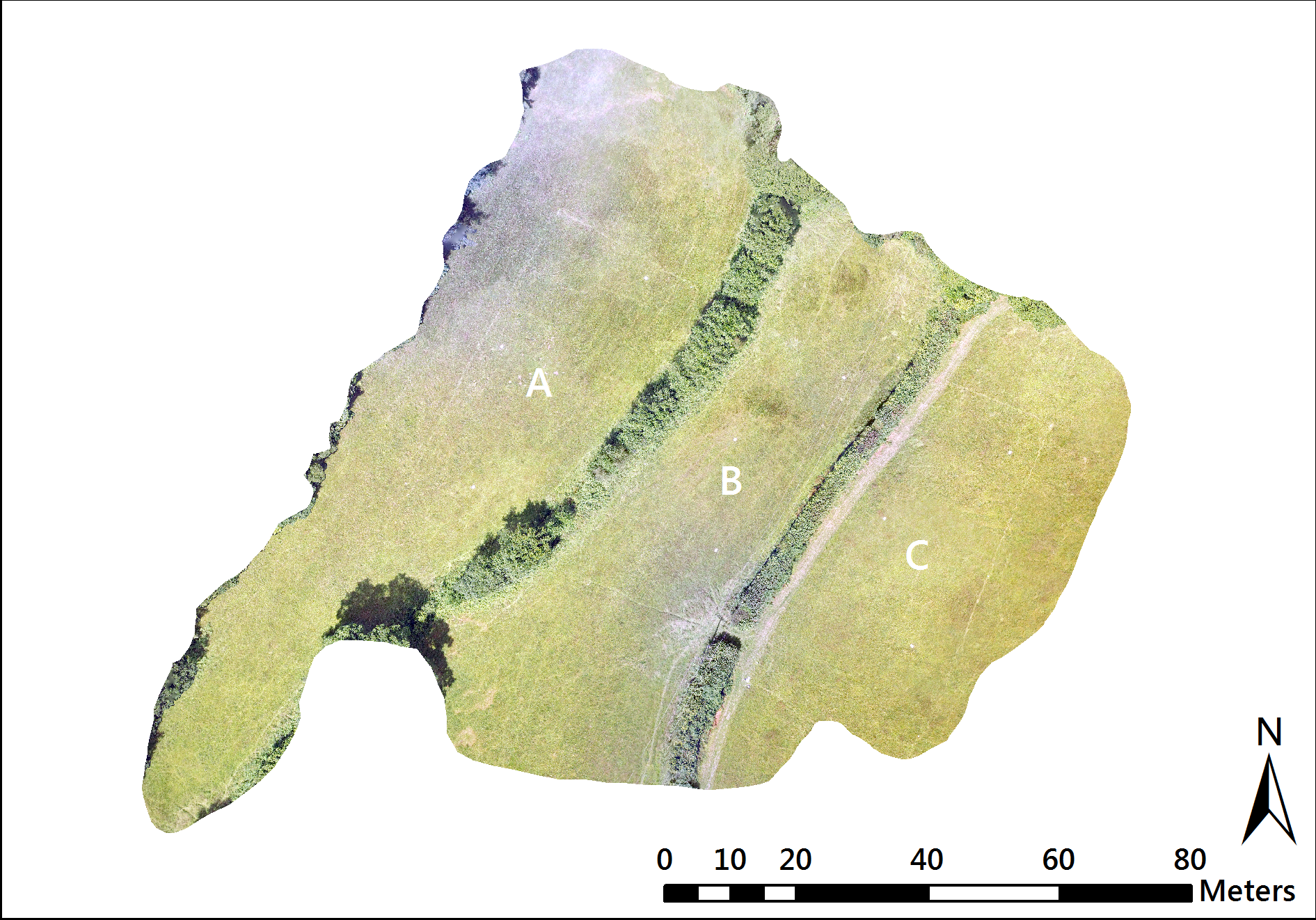

Supplement: S1 Fig — Fields A and B = MG5c unimproved grassland, field C = MG6b improved grassland. (TIF) [file pone.0186193.s001.tif]
