# Supplementary material for: The potential of small-Unmanned Aircraft Systems for the rapid detection of threatened unimproved grassland communities using an Enhanced Normalized Difference Vegetation Index
Source: PLoS One. 2017 Oct 12;12(10):e0186193. doi: 10.1371/journal.pone.0186193 (PMC5638390; doi:10.1371/journal.pone.0186193)
Supplement: S1 Table — (DOCX) [file pone.0186193.s002.docx]

Table S1. A summary of mean species values, standard deviation (S.D.) and 95% confidence intervals (CI) for the vegetation survey data.

|  |  | | **Unimproved grassland (MG5c)** | **Improved grassland (MG6b)** | **Rush pasture (M23)** |
| --- | --- | --- | --- | --- | --- |
| **Total species** | **Mean:** | | 15.830 | 10.000 | 15.500 |
|  | **S.D:** | | 2.995 | 2.000 | 5.000 |
|  | **95% CI:** | **Lower bound:** | 13.890 | 8.060 | 11.380 |
|  |  | **Upper bound:** | 17.780 | 11.940 | 19.620 |
| **Grass species** | **Mean:** | | 3.555 | 4.780 | 2.500 |
|  | **S.D:** | | 0.735 | 0.830 | 0.710 |
|  | **95% CI:** | **Lower bound:** | 3.015 | 4.240 | 1.350 |
|  |  | **Upper bound:** | 4.100 | 5.320 | 3.650 |
| **Wildflower species** | **Mean:** | | 10.500 | 4.220 | 10.000 |
|  | **S.D:** | | 2.145 | 1.920 | 4.240 |
|  | **95% CI:** | **Lower bound:** | 8.990 | 2.710 | 6.790 |
|  |  | **Upper bound:** | 12.010 | 5.730 | 13.210 |
| **Rush and sedge species** | **Mean:** | | 0.555 | 0.000 | 2.000 |
|  | **S.D:** | | 0.770 | 0.000 | 0.000 |
|  | **95% CI:** | **Lower bound:** | 0.130 | -0.420 | 1.100 |
|  |  | **Upper bound:** | 0.980 | 0.420 | 2.900 |
| **Tree (sapling) species)** | **Mean:** | | 0.220 | 0.000 | 0.000 |
|  | **S.D:** | | 0.520 | 0.000 | 0.000 |
|  | **95% CI:** | **Lower bound:** | -0.080 | -0.300 | -0.640 |
|  |  | **Upper bound:** | 0.525 | 0.300 | 0.640 |
