# Supplementary material for: The potential of small-Unmanned Aircraft Systems for the rapid detection of threatened unimproved grassland communities using an Enhanced Normalized Difference Vegetation Index
Source: PLoS One. 2017 Oct 12;12(10):e0186193. doi: 10.1371/journal.pone.0186193 (PMC5638390; doi:10.1371/journal.pone.0186193)
Supplement: S2 Table — (DOCX) [file pone.0186193.s003.docx]

Table S1. Vegetation index statistics (Min value, max value, mean, standard deviation) for each of the three habitat communities present within the study site.

|  | **NDVI** | | | | **GNDVI** | | | | **GDVI** | | | |
| --- | --- | --- | --- | --- | --- | --- | --- | --- | --- | --- | --- | --- |
| **Field** | **Min** | **Max** | **Mean** | **S.D.** | **Min** | **Max** | **Mean** | **S.D.** | **Min** | **Max** | **Mean** | **S.D.** |
| **Improved grassland (MG6b)** | 0.37636 | 0.69532 | 0.51247 | 0.04456 | 0.25661 | 0.50159 | 0.37043 | 0.03401 | 95.44444 | 124.77778 | 111.99865 | 3.71492 |
| **Unimproved grassland (MG5c)** | 0.32865 | 0.65421 | 0.49805 | 0.04127 | 0.23531 | 0.49125 | 0.37243 | 0.03212 | 90.50000 | 127.33333 | 115.66133 | 4.08536 |
| **Rush pasture (M23)** | 0.40418 | 0.67192 | 0.50994 | 0.04460 | 0.23560 | 0.51405 | 0.38501 | 0.03433 | 88.83951 | 128.56790 | 117.22040 | 4.43188 |
|  | | | | | | | | | | | | |
|  | **GIPVI** | | | | **GRVI** | | | | **ENDVI** | | | |
| **Field** | **Min** | **Max** | **Mean** | **S.D.** | **Min** | **Max** | **Mean** | **S.D.** | **Min** | **Max** | **Mean** | **S.D.** |
| **Improved grassland (MG6b)** | 0.62831 | 0.75080 | 0.68521 | 0.01701 | 1.69657 | 3.02407 | 2.18825 | 0.17619 | 0.27175 | 0.57782 | 0.39011 | 0.04223 |
| **Unimproved grassland (MG5c)** | 0.61961 | 0.74652 | 0.68611 | 0.01608 | 1.63985 | 2.95943 | 2.19765 | 0.16806 | 0.22437 | 0.52968 | 0.37108 | 0.03883 |
| **Rush pasture (M23)** | 0.61823 | 0.75722 | 0.69248 | 0.01717 | 1.63106 | 3.19504 | 2.27560 | 0.18954 | 0.21858 | 0.54978 | 0.38170 | 0.04255 |
